# A 3D contact analysis approach for the visualization of the electrical contact asperities

**DOI:** 10.1063/1.4974151

**Published:** 2017-01-11

**Authors:** Constantinos C. Roussos, Jonathan Swingler

**Affiliations:** School of Engineering and Physical Sciences, Heriot-Watt University, Edinburgh EH14 4AS, United Kingdom

## Abstract

The electrical contact is an important phenomenon that should be given into consideration to achieve better performance and long term reliability for the design of devices. Based upon this importance, the electrical contact interface has been visualized as a ‘‘3D Contact Map’’ and used in order to investigate the contact asperities. The contact asperities describe the structures above and below the contact spots (the contact spots define the 3D contact map) to the two conductors which make the contact system. The contact asperities require the discretization of the 3D microstructures of the contact system into voxels. A contact analysis approach has been developed and introduced in this paper which shows the way to the 3D visualization of the contact asperities of a given contact system. For the discretization of 3D microstructure of contact system into voxels, X-ray Computed Tomography (CT) method is used in order to collect the data of a 250 *V*, 16 *A* rated AC single pole rocker switch which is used as a contact system for investigation.

## I. INTRODUCTION

The nature of real flat surfaces of solid bodies which seem to be flat at first sight in macroscale in reality are rough at the microscale and further rough in nanoscale.^1–4^ When the surfaces of the two bodies are brought together their roughness influence mechanical contact which occurs only in a specific number of areas on the apparent area of contact. The roughness of each surface consists of peak and valleys whose shape, height variation, average separation and other geometrical characteristics depend on the manufacturing process and material used. The peaks of the roughness of surfaces which are in mechanical contact are called contact spots and their structures above and below the two bodies are called contact asperities.

The contact spots are found to be very important by many researchers and visualized using different methods. The visualization methods can be classified into destructive and non-destructive.^5^ Destructive methods such as Thermo-Graphic (TG)^6^ and Scanning Electron Microscopy (SEM)^7^ can be applied if one part of the surface is replaced to enable the viewing of the surface, or if both bodies of the original contact are inspected, are necessary to be dismantled after testing for analysis. Non-destructive methods such as Magnetic Resonance Imaging (MRI)^8,9^ and X-ray Computed Tomography (CT)^5,10–14^ are of more interest because they offer the opportunity to acquire 2D and 3D views of the samples without dismantling the component parts and thus destroy any features of interest. In addition to MRI and X-ray CT, there are different numerical approaches to show the contact spots.^15,16^

Many recent contact spots visualization methods do not recognize the effects of scale dependent properties;^6,10,17,18^ in fact, many classical and widely used contact spots area visualization methods completely ignore the effect of scale and the 3D nature of contact spots and picture the contact area in a 2D plane. This current work aims to build on the visualization method developed in previous work^11^ where the contact spots are pictured in 3D plane as a ‘‘3D Contact Map’’.

The contact asperities has been investigated for decades due to their significant importance in several branches of science and engineering such as surface science,^19–21^ tribology,^22–24^ heat transfer^25–28^ and recently in Micro-Electro-Mechanical Systems (MEMS).^29–35^ Due to this significant importance, several models^36–41^ are developed in order to provide information about their features such as contact asperity dimensions, number, distribution material properties, surface profiles and operating conditions. One of the most popular models has been developed by Greenwood-Williamson.^42^ According to this model, it assumes the contact asperities on a surface are hemispherical in shape with the same radius. The peak of each contact asperity is assumed to be located at different heights following a random Gaussian distribution. When a flat plane is brought into contact with the Greenwood-Williamson surface, the contact asperities deform elastically with consideration of plastic deformation under particular limits.

In this current work, a contact analysis approach has been developed and introduced which shows the structures of the contact spots of the conductors of a 250 *V*, 16 *A* rated AC single pole rocker switch as 3D contact asperities. It is important to note that this current work is based on the visualization method developed in Ref. 11 which the contact spots are pictured as 3D contact map. Moreover, the volume and surface area exposed to air of each 3D contact asperity are calculated and presented with their distribution.

## II. EXPERIMENTAL DETAILS

### A. Contact system investigation and macro-visualization

**FIG. 1.**
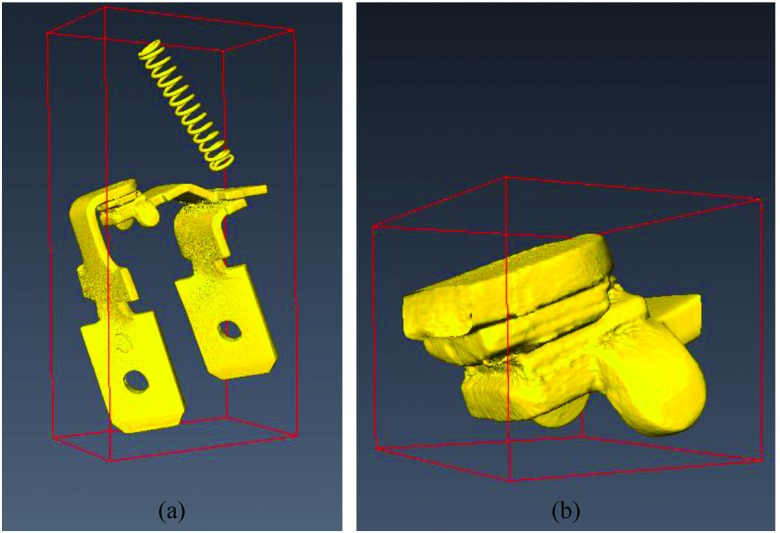
(a) Macro visualization of internal view of the single pole rocker switch, (b) Closed-up view of the volume of interest.

A 250 *V*, 16 *A* rated AC single pole rocker switch with dimensions (3.0 x 2.5 x 3.5) c*m* is used as a contact system for investigation. The contact material consists of silver alloy while other conductors are made of copper alloy. The internal view of the metalwork of the single pole rocker switch is presented in Fig. 1a. It consists of contact force spring and conductors. The geometry of the contact pair is a flat on flat with surface roughness (*R*_*a*_) measured to be 0.42 ± 0.11 μ*m* for Conductor A and 0.25 ± 0.04 μ*m* for Conductor B. The surface roughness test was carried out using a contact profilometer Taylor-Hobson RTH Talysurf 5-120 with a lateral *x* resolution of 0.1 μ*m* and height *y* resolution of 0.1 n*m*. Moreover, the force (*F*) of the contact force spring is measured to be 1.89 ± 0.07 *N*.^11^ Fig. 1b shows the closed-up view of the contact pair of the two conductors which is the volume of interest.

### B. X-ray CT visualization method

The X-ray CT visualization method consists of several stages starting with acquiring X-ray images of the contact system using an HMX 225 μCT system scanner which operates using an X-ray tomography designed by the XTek Group. The X-ray source is set to 175 k*V*, 133 μA which gives 3 μm focus capability. The scanner rotates the contact system through 360°, taking a series of 2D X-ray images (2439 images are taken).

The second stage is the reconstruction of the 2D X-ray images to 3D reconstructed model of the contact system using the ‘‘CT-Pro’’ software. This 3D reconstructed model is used for all subsequent analysis of the data. The 2D X-ray images are 16-bit grayscale images which specify the level of X-ray absorption through the contact system at different angles. Consequently, each 2D X-ray image contains 3D information of the contact system at particular angles to the X-ray beam direction.^1^ The ‘‘CT-Pro’’ software amalgamates all these 2D X-ray images taken across the 360° around the contact system by using the cone beam back-projection technique to form the 3D reconstructed model of the contact system reconstruction. Each voxel within the 3D reconstructed model of the contact system has a grayscale value indicating the level of X-ray absorption and consequently the material density.

**FIG. 2.**
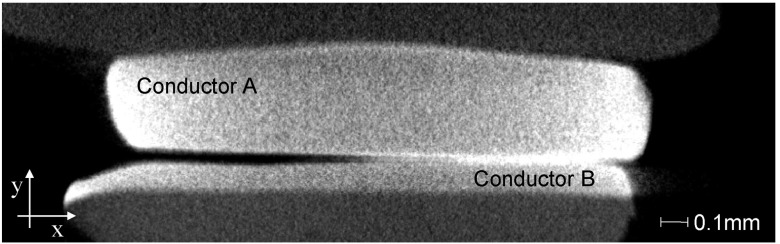
16-bit 2D cross-section slice image.

The third stage is the use of the ‘‘VGStudioMax’’ software in order to create 16-bit 2D cross-section slice images from the 3D reconstructed model of the contact system which gives multiple cross-section views of the contact system. This software separates the x-y-z volume of the 3D reconstructed model of the contact system into y number of x-z 16-bit 2D cross-section slice images. Fig. 2 shows an example of a 16-bit 2D cross-section slice image from the 3D contact pair of the two conductors of Fig. 1b. The various intensities of pixel illuminations related to the level of X-ray absorption indicate different materials within a voxel. The more highly absorbing silver alloy (lighter greyscale) is indicated with the less absorbing copper alloy metal, compared to minimally absorbing air (black on the greyscale). The darker region between the two conductors is indicating an air gap.

These 16-bit 2D cross-section slice images are converted to 1-bit images in order to separate the metal parts (white areas) of the contact system from the air (black areas) as explained in previous work.^5^ In this paper, the 1-bit 2D cross-section slice images of the contact system are analyzed with Contact Analysis Techniques (CAT*) which are developed and implemented with a suite of tools developed in MATLAB and Image Processing Toolbox. These CAT* are developed in order to visualize the electrical contact asperities of the 250 *V*, 16 *A* rated AC single pole switch.

## III. CONTACT ANALYSIS AND MODELING APPROACH

### A. The concept and characteristics of a contact system

**FIG. 3.**
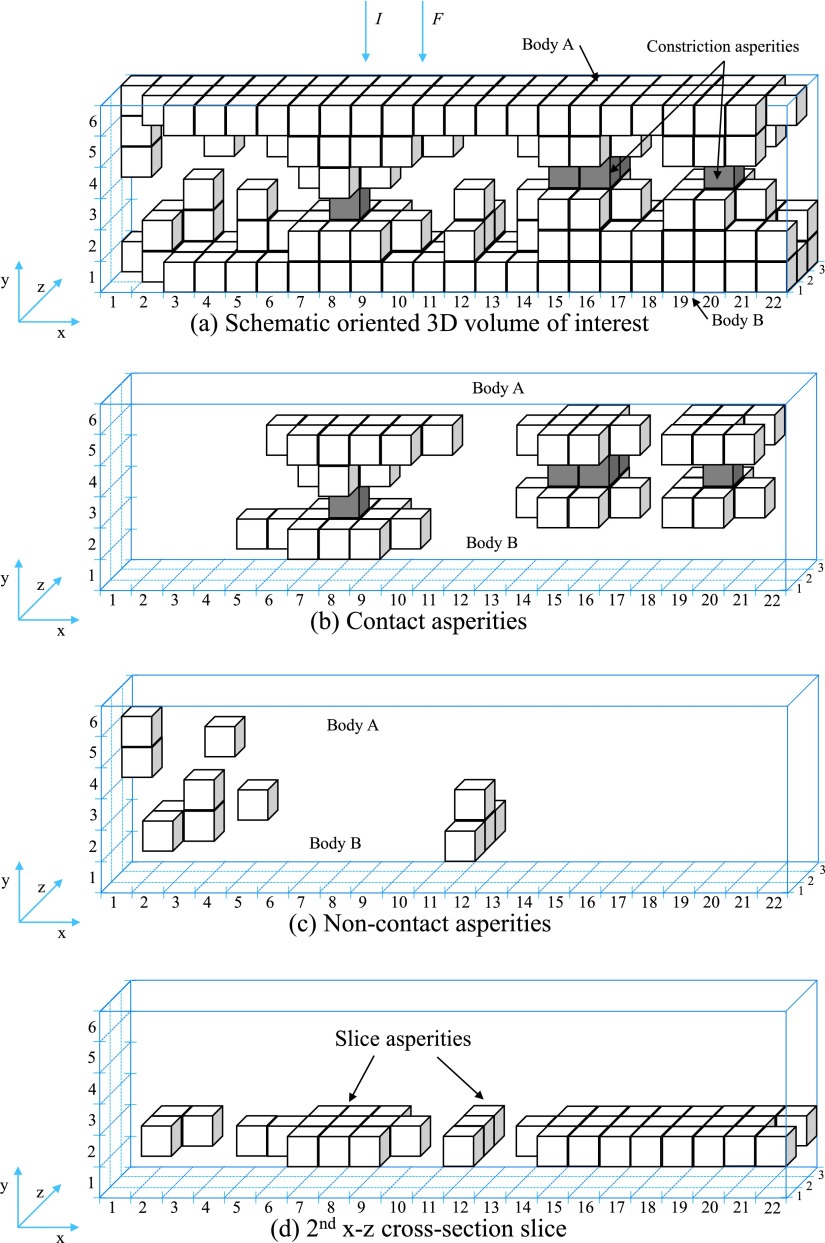
Schematic contact system with its characteristics.

For the 3D visualization of contact asperities, a similar approach was used in the previous work^14^ in order to picture any cross-section slice of the contact system showing from which voxels the electric current flows is used. Fig. 3a shows a schematic oriented 3D volume of interest of a contact system which is used in order to explain this contact analysis approach. It consists of two rough bodies, A and B which are in mechanical contact. The mechanical contact occurs at the three constriction asperities (groups of grey voxels in Fig. 3). In this research, the structures of these constriction asperities above and below the two bodies of the schematic oriented 3D volume of interest of a contact system are called contact asperities while the roughness of two bodies which their ‘‘peaks’’ are not in contact are called non-contact asperities. The schematic oriented 3D volume of interest of a contact system of Fig. 3a consists of 3 contact asperities and 5 non-contact asperities (2 for the Body A and 3 for the Body B). These asperities (contact asperities and non-contact asperities) are illustrated in Fig. 3b and Fig. 3c respectively. It is important to note that the number of contact asperities for both bodies A and B of any contact system is equal. The schematic contact system of Fig. 3a consists of 6 x-z cross-section slices. The 2^nd^ x-z cross-section slice of Fig. 3a which consists of 4 slice asperities is illustrated in Fig. 3d. A slice asperity is defined as a collection of voxels which are neighboring other voxels by at least one point of their edges.

### B. The contact analysis approach for the asperities visualization

The contact analysis approach consists of further stages starting with the division of the contact system into equal x-z cross-section slices across the electric current (*I*) direction (y-direction). The electric current direction is defined to be parallel with the normal force (*F*) and it is assumed that it flows through the whole cross-section area of the first and last x-z cross-section slices. The direction of the normal force is used to define the orientation of the coordinate system used.

**FIG. 4.**
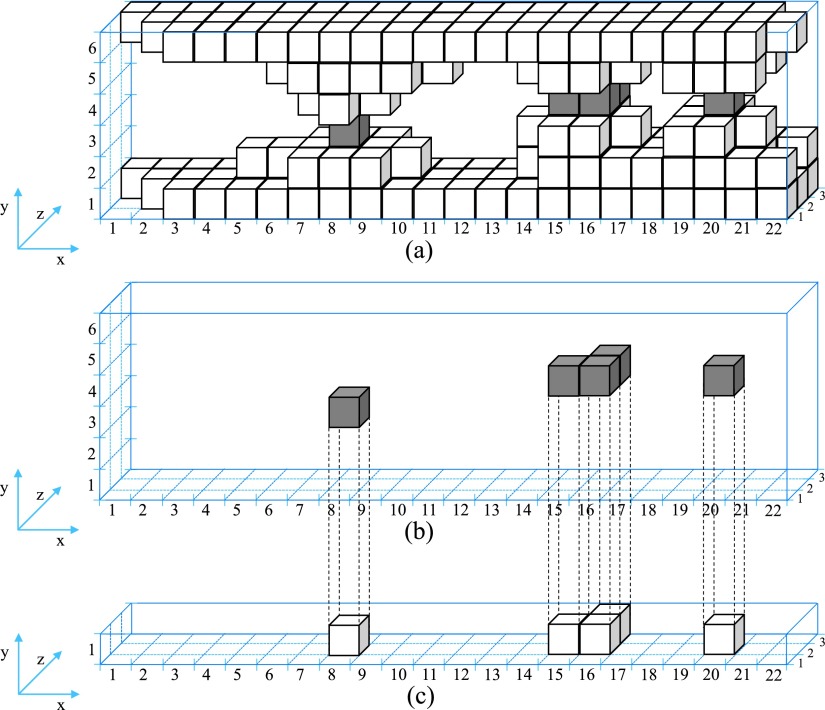
Schematic (a) 3D contact source model, (b) 3D constriction asperities map, (c) x-z contact slice.

The second stage of the contact analysis approach is the development of the 3D contact source model of the contact system which is illustrated in Fig. 4a. This model includes only the contact asperities from which the electric current flows when a potential difference is applied across the two bodies, A and B. For example, Fig. 4a includes only the contact asperities of Fig. 3b with their full structures to the two bodies, A and B. More details about the development of the 3D contact source model of the contact system are given in previous work.^14^

To visualize only the contact asperities of the 3D contact source model of the contact system of Fig. 4a three Contact Analysis Techniques (CAT*) are developed. The first technique is to develop the 3D constriction asperities map using the Contact Analysis Technique for Asperities (CATA) which gives information on where the constriction asperities in a 3D volume profile are located. CATA shows that the electric current flows through the 3D contact asperities map.^13^ This technique is a continuation of the 3D contact maps developed in previous work^11^ and extended by one voxel in electric current direction as presented in Ref. 13. Fig. 4b illustrates the 3D constriction asperities of the schematic 3D contact source model of the contact system of Fig. 4a.

The second technique is the Contact Analysis Technique for Contact Voxels (CATV). This technique is used to create an x-z contact slice with all the constriction asperities at the same height (y-direction) as illustrated in Fig. 4c. As mentioned before, the electric current flows through the 3D constriction asperities map, consequently, it flows through the x-z contact slice. The collection of solid voxels in this x-z contact slice which are neighboring other solid voxels by at least one point of their edges are defined as slice asperities (same definition as in x-z cross-section slices). The x-z contact slice of Fig. 4c consists of 3 slice asperities.

The third technique, Contact Analysis Technique for Asperities Comparison (CATAC) which consists of several stages starts with the visualization of each slice asperity separately with its structures to the two bodies A and B. To achieve this, a comparison of each slice asperity of the x-z contact slice with the slice asperities of each x-z cross-section slice is made. The reason of making a comparison is to identify which of the slice asperities of the x-z cross-section slice are connected with the slice asperity *k* of the x-z contact slice. Where *k*, is the number of slice asperity of the x-z contact slice (it is also the number of contact asperity, as the slice asperity belongs to the constriction asperity). If there is a connection between the slice asperity *k* of the x-z contact slice with any of the slice asperities in the x-z cross-section slice, then, the connected slice asperity in the x-z cross-section slice belongs to the slice asperity *k*. If there is no connection between the slice asperity *k* of the x-z contact slice with the slice asperities in the x-z cross-section slice, then, the disconnected slice asperities are removed from the x-z cross-section slice. A mathematical example of this technique is given below describing the 3D visualization of contact asperities of the schematic 3D contact source model of Fig. 4a where each x-z cross-section slice is described by different matrix.

The matrix [A] of Eq. (3) represents the x-z contact slice of Fig. 4c, where zeros and *a* elements of matrix [A] represent voxels of air and solid material of the schematic 3D contact source model of contact system respectively. A slice asperity in matrix [A] is defined as a collection of solid voxels which are neighboring other solid voxels by at least one point of their edges. The matrix [A], or the x-z contact slice consists of three slice asperities.

$$\left[A\right]=\left[\begin{array}{cccccccccccccccccccccc} 0 & 0 & 0 & 0 & 0 & 0 & 0 & 0 & 0 & 0 & 0 & 0 & 0 & 0 & 0 & \alpha & 0 & 0 & 0 & 0 & 0 & 0\\ 0 & 0 & 0 & 0 & 0 & 0 & 0 & \alpha & 0 & 0 & 0 & 0 & 0 & 0 & \alpha & \alpha & 0 & 0 & 0 & \alpha & 0 & 0\\ 0 & 0 & 0 & 0 & 0 & 0 & 0 & 0 & 0 & 0 & 0 & 0 & 0 & 0 & 0 & 0 & 0 & 0 & 0 & 0 & 0 & 0 \end{array}\right]$$ (1)

Matrix $\left[A_{\text{k}}\right]$, represents the *k* slice asperity of the x-z contact slice for k∈[1,S]. Where *S*, is the total number of slice asperities of the x-z contact slice (or the total number of slice asperities of matrix [A]). The matrix $\left[A_{1}\right]$ in Eq. (2) represents the 1^st^ slice asperity of the x-z contact slice.

$$\left[A_{1}\right]=\left[\begin{array}{cccccccccccccccccccccc} 0 & 0 & 0 & 0 & 0 & 0 & 0 & 0 & 0 & 0 & 0 & 0 & 0 & 0 & 0 & 0 & 0 & 0 & 0 & 0 & 0 & 0\\ 0 & 0 & 0 & 0 & 0 & 0 & 0 & \alpha & 0 & 0 & 0 & 0 & 0 & 0 & 0 & 0 & 0 & 0 & 0 & 0 & 0 & 0\\ 0 & 0 & 0 & 0 & 0 & 0 & 0 & 0 & 0 & 0 & 0 & 0 & 0 & 0 & 0 & 0 & 0 & 0 & 0 & 0 & 0 & 0 \end{array}\right]$$ (2)

Matrix $\left[B_{i}\right]$, represents the *i* x-z cross-section slice for i∈[1,N]. Where *i*, is the number of x-z cross-section slice and *N* is the total number of x-z cross-section slices. Zeros and *β* elements of matrix $\left[B_{i}\right]$ represent voxels of air and solid material of the 3D contact source model of contact system respectively. The collection of solid voxels in matrix $\left[B_{i}\right]$ which are neighboring to other solid voxels by at least one point of their edges are called slice asperities. The matrix $\left[B_{2}\right]$, or the 2^nd^ x-z cross-section slice of Fig. 4a consists of 2 slice asperities and is described by Eq. (3).

$$\left[B_{2}\right]=\left[\begin{array}{cccccccccccccccccccccc} 0 & 0 & 0 & 0 & 0 & 0 & \beta & \beta & \beta & 0 & 0 & 0 & 0 & 0 & \beta & \beta & \beta & \beta & \beta & \beta & \beta & \beta \\ 0 & 0 & 0 & 0 & \beta & \beta & \beta & \beta & \beta & \beta & 0 & 0 & 0 & \beta & \beta & \beta & \beta & \beta & \beta & \beta & \beta & 0\\ 0 & 0 & 0 & 0 & 0 & 0 & \beta & \beta & \beta & 0 & 0 & 0 & 0 & 0 & \beta & \beta & \beta & \beta & \beta & \beta & \beta & \beta \end{array}\right]$$ (3)

To identify if there is a connection between the 1^st^ (k=1) slice asperity of x-z contact slice with any of the slice asperities in the 2^nd^ (i=2) x-z cross-section slice, Eqs. (2) and (3) are added as presented in Eq. (4). The matrix $\left[C_{k_{i}}\right]$ is the sum of matrix $\left[A_{k}\right]$ with matrix $\left[B_{i}\right]$. The *γ* element represents the summation of *a* and *β* elements and shows if there is a connection between the slice asperity *k* of the x-z contact slice with any of the slice asperities in the *i* x-z cross-section slice. The same procedure is used for the rest of the x-z cross-section slices.

$$\left[C_{1_{2}}\right]=\left[\begin{array}{cccccccccccccccccccccc} 0 & 0 & 0 & 0 & 0 & 0 & \beta & \beta & \beta & 0 & 0 & 0 & 0 & 0 & \beta & \beta & \beta & \beta & \beta & \beta & \beta & \beta \\ 0 & 0 & 0 & 0 & \beta & \beta & \beta & \gamma & \beta & \beta & 0 & 0 & 0 & \beta & \beta & \beta & \beta & \beta & \beta & \beta & \beta & 0\\ 0 & 0 & 0 & 0 & 0 & 0 & \beta & \beta & \beta & 0 & 0 & 0 & 0 & 0 & \beta & \beta & \beta & \beta & \beta & \beta & \beta & \beta \end{array}\right]$$ (4)

Each of the slice asperities presented in the matrix $\left[C_{k_{i}}\right]$ is examined separately in order to identify if it belongs to the *k* 3D contact asperity with its structures to bodies A and B. If a slice asperity belongs to this *k* 3D contact asperity with its structures to bodies A and B, the *γ* element is included within the slice asperity and a new matrix is created which contains only this slice asperity which is renamed with the *δ* elements. A slice asperity without the *γ* element is replaced with zeros. These conditions are described by matrix $\left[D_{{\text{k}}_{i}}\right]$. The matrix $\left[D_{1_{2}}\right]$ of Eq. (5) shows that the slice asperity (*δ* elements) of the 2^nd^ (i=2) x-z cross-section slice of matrix [D] belongs to the 1^st^ (k=1) 3D contact asperity with its structures to bodies A and B. The same procedure is used for the rest of the x-z cross-section slices of the 3D contact source model.

$$\left[D_{1_{2}}\right]=\left[\begin{array}{cccccccccccccccccccccc} 0 & 0 & 0 & 0 & 0 & 0 & \delta & \delta & \delta & 0 & 0 & 0 & 0 & 0 & 0 & 0 & 0 & 0 & 0 & 0 & 0 & 0\\ 0 & 0 & 0 & 0 & \delta & \delta & \delta & \delta & \delta & \delta & 0 & 0 & 0 & 0 & 0 & 0 & 0 & 0 & 0 & 0 & 0 & 0\\ 0 & 0 & 0 & 0 & 0 & 0 & \delta & \delta & \delta & 0 & 0 & 0 & 0 & 0 & 0 & 0 & 0 & 0 & 0 & 0 & 0 & 0 \end{array}\right]$$ (5)

**FIG. 5.**
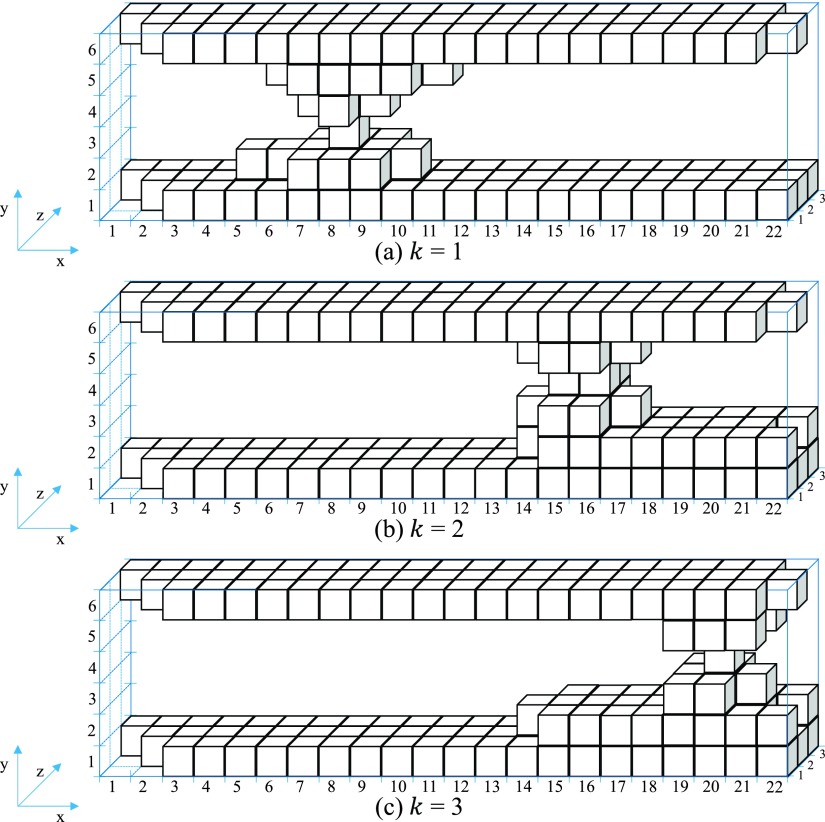
3D contact asperities with their structures to bodies A and B.

Fig. 5a shows the 1^st^ (k=1) 3D contact asperity with its structures to bodies A and B which is developed with the stack of matrices $\left[D_{1_{i}}\right]$ in the y-direction for i∈[1,N]. The same procedure used for the visualization of 1^st^ (k=1) 3D contact asperity with its structures to bodies A and B is used for the rest of *k* 3D contact asperities with their structures to bodies A and B. The results of this procedure for the 2^nd^ and 3^rd^ (k=2 and k=3) 3D contact asperities with their structures to bodies A and B are illustrated in Fig. 5b and Fig. 5c respectively.

For the visualization of actual 3D contact asperities (without their full structures to bodies A and B) the 3D contact asperities with their structures to bodies A and B presented in Fig. 5 are used to create 3D matrices for examination. Each 3D matrix $\left[E_{k}\right]$ represents the *k* 3D contact asperity with its structures to bodies A and B of Fig. 5. Each voxel of the solid of Fig. 5 represented with ε element in the $\left[E_{k}\right]$ 3D matrix while the air is represented with zero elements. For the separation of 3D contact asperities from their full structures to bodies A and B, all the 3D matrices, $\left[E_{k}\right]$ are added as described in Eq. (6).

$$\left[F\right]=\sum_{k=1}^{S}\left[E_{k}\right].$$ (6)

**FIG. 6.**
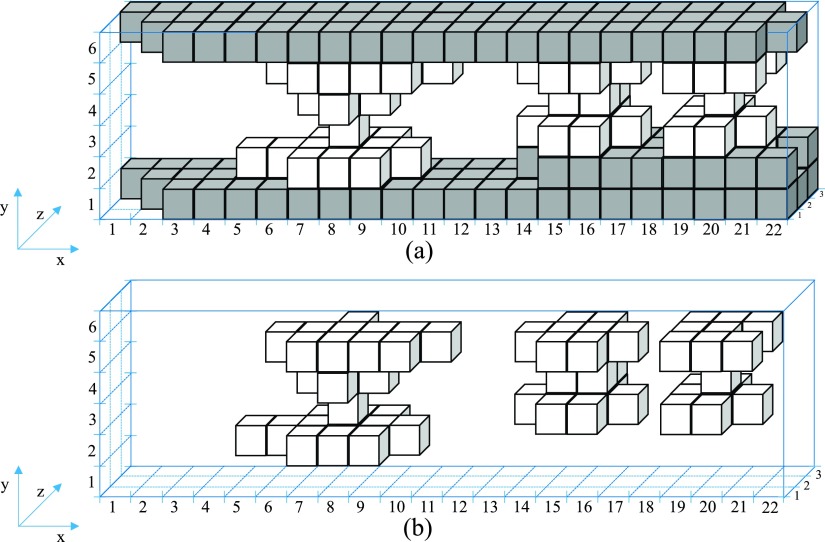
(a) Summation of the voxels of 3D contact asperities with their structures to bodies A and B, (b) 3D contact asperities.

The summation of Eq. (6) is illustrated in Fig. 6a with voxels in different colors. The color of each voxel depends on the value of each element $\varphi_{x,y,z}$ (where the suffixes *x*,*y*,*z* represent the position of the *φ* element in the 3D matrix [F]) of the 3D matrix [F]. The $\varphi_{x,y,z}$ element takes three types of values as described from Eq. (7). The elements with zero value represent the air while the elements with ε and m⋅ε values represent white and gray voxels respectively. The zero values of the 3D matrix [F] are not illustrated in the figures as they represent air.

$$\varphi_{x,y,z}=\left\{ \begin{array}{c} 0\\ \varepsilon \\ m\cdot \varepsilon ,\;\left(m\in \mathbb{R}\right) \end{array}.\right.$$ (7)

The final stage of the CATAC technique is to visualize only the 3D contact asperities (white voxels in Fig. 6a). At this stage each element $\varphi_{x,y,z}$ in the 3D matrix [F] of Eq. (6) is examined separately as described from Eq. (8). If $\varphi_{x,y,z}=\varepsilon$ then the $g_{x,y,z}$ element in the 3D matrix [G] equals to *g* and if $\varphi_{x,y,z}\neq \varepsilon$, then the $g_{x,y,z}$ element equals to zero. The zero and *g* values in 3D matrix [G] represent air and solid respectively. The result of the 3D matrix [G] in voxels is illustrated in Fig. 6b. The collection of solid voxels which are neighboring to other solid voxels by at least one point of their edges are called 3D contact asperities.

$$g_{x,y,z}=\left\{ \begin{array}{cc} g,\;if\varphi_{x,y,z}=\varepsilon & \\ 0,\;if\varphi_{x,y,z}\neq \varepsilon & \end{array}\right..$$ (8)

## IV. RESULTS AND ANALYSIS

### A. Contact system

**FIG. 7.**
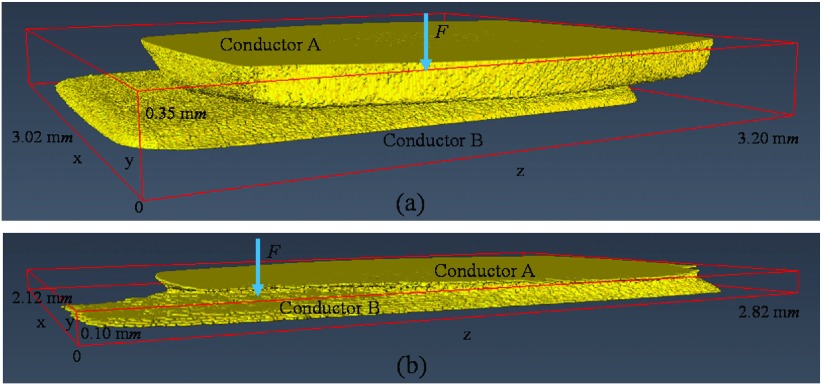
(a) 3D source model of the contact system, (b) 3D contact source model of the contact system.

Fig. 7a illustrates a part of the 3D volume of interest of contact system of the 250 *V*, 16 *A* rated AC single pole rocker switch which is labeled as a 3D source model. This part of the volume with voxel resolution of 5 μ*m* × 5 μ*m* × 5 μ*m* is selected from the 3D volume of interest presented in Fig. 1b and oriented so that its normal force (*F*) to be parallel with y-axis (the reason is given in Section III.B). More details concerning the selection of this part of volume (3D source model) from the 3D volume of interest of Fig. 1b are given in previous work.^14^

Fig. 7b shows the 3D contact source model of the contact system of Fig. 7a. The 3D contact source model is visualized using the 2D cross-section slice images of 3D source model which have been processed as described in previous work.^14^ It is important to note that for the 3D contact source model visualization only a part of 2D cross-section slice images of 3D source model are used and the reason is explained in Section V. The distances between the first and last x-z cross-section slices of the contact systems of Fig. 7a and Fig. 7b (y-direction) are calculated to be 68 pixels length (0.34 m*m*) and 18 pixels length (0.09 m*m*) respectively.

### B. 3D contact map and x-z contact slice

**FIG. 8.**
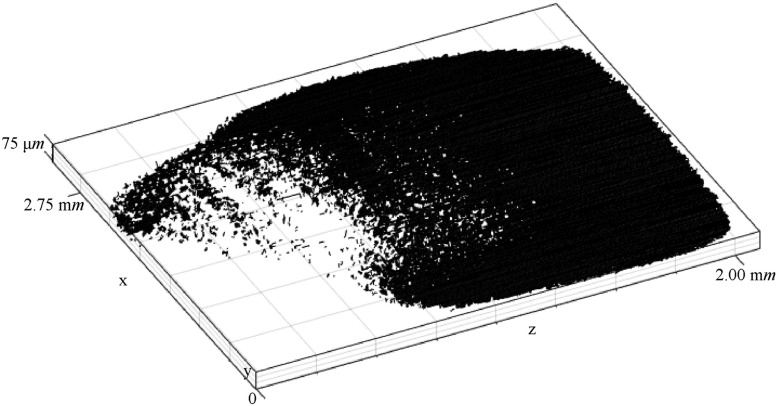
3D contact map.

Fig. 8 shows the 3D contact map of the contacting interface between the conductors of the contact system of Fig. 7. The 3D contact map is visualized using the 2D cross-section slice images which are processed and implemented using CAT* with a suite of tools developed in MATLAB as described in previous work.^11^ This map, consists of contact spots (pixels, surfaces) which are the cross-section areas of the 3D constriction asperities map (voxels, volumes).

**FIG. 9.**
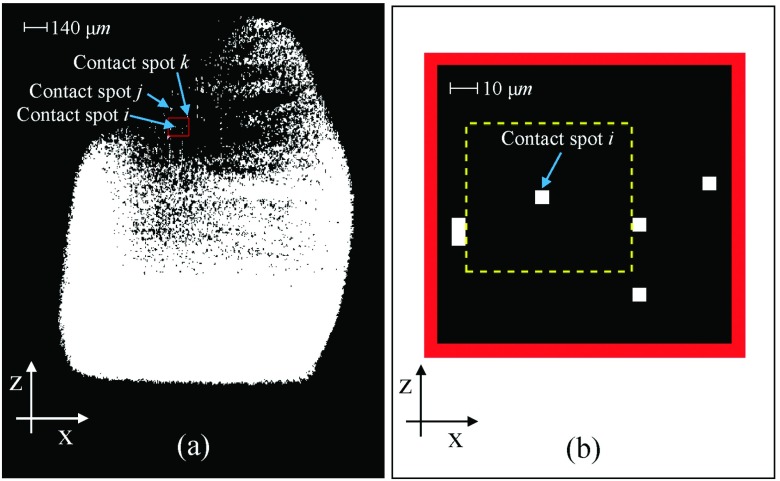
(a) 2D contact map of 3D contact source model of the contact system, (b) Closed-up view of the red box of 2D contact map.

Fig. 9a shows the cross-section of the x-z contact slice of the contact system of Fig. 7. The x-z contact slice is developed using CATV which all the contact spots of the 3D contact map of Fig. 8 are set to the same height (y-direction). The cross-section contact slice of Fig. 9a is also called 2D contact map. Fig. 9b shows the closed-up view of the red box of 2D contact map of Fig. 9a which includes 5 contact spots.

The yellow dash-box of Fig. 9b illustrates the area which is cropped in each x-z cross-section slice around the contact spot *i* with its full structures to two conductors of Fig. 11a across the y-direction. The stack of these cropped x-z cross-section slices around the contact spot *i* with its full structures to two conductors across the y-direction is illustrated as volume of interest around contact spot *i* in Fig. 11b.

### C. 3D contact asperities

**FIG. 10.**
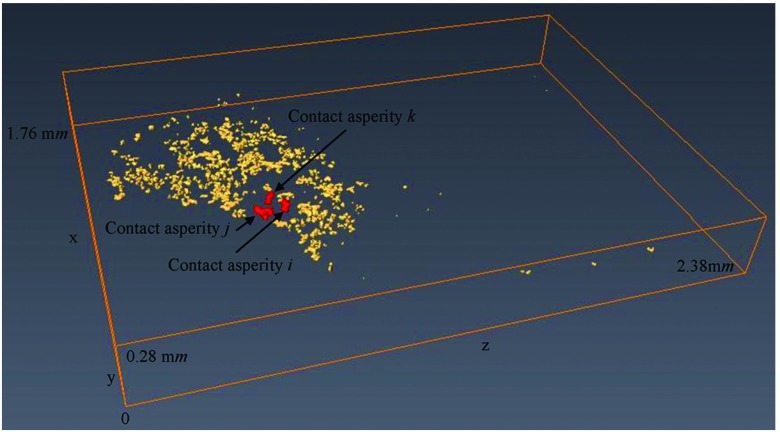
3D contact asperities.

Fig. 10 shows the 3D contact asperities of the contact system of Fig. 7. The largest 3D contact asperity of the contact system (the structures above and below the largest contact spot in the 2D contact map of Fig. 9a) is not presented in Fig. 10 because it hides the rest of the 3D contact asperities.

**FIG. 11.**
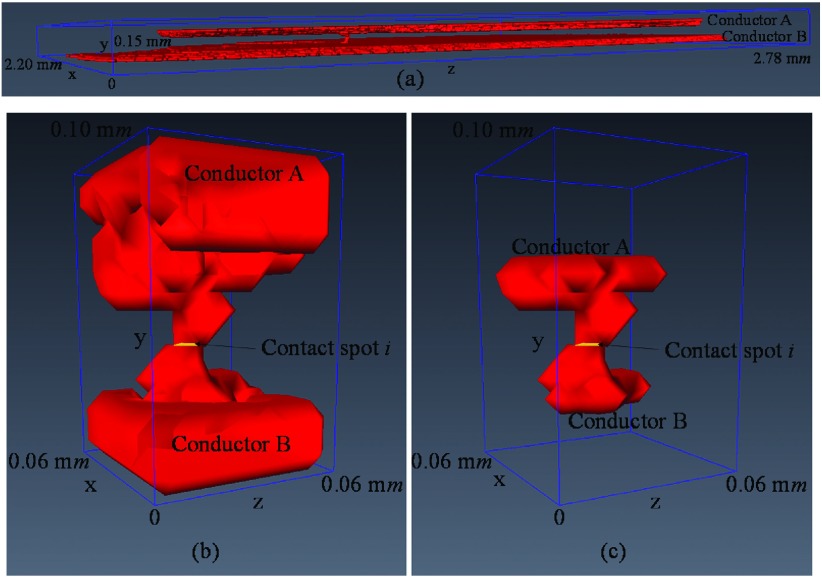
(a) Contact spot i with its full structures to conductors, (b) Volume of interest around contact spot *i* and (c) Contact asperity *i*.

**FIG. 12.**
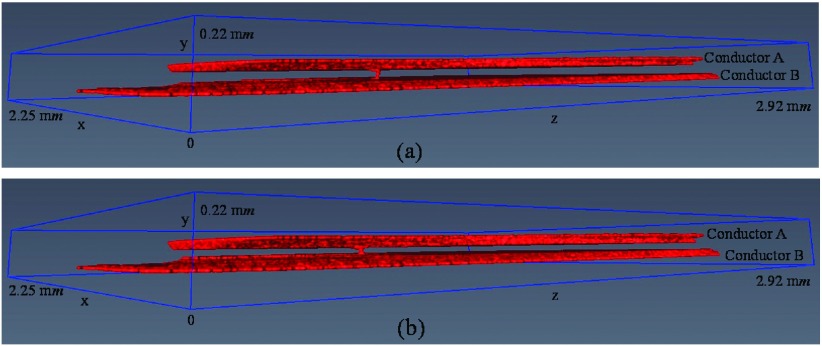
(a) Contact spot *j* with its full structures to conductors and (b) Contact spot *k* with its full structures to conductors.

**FIG. 13.**
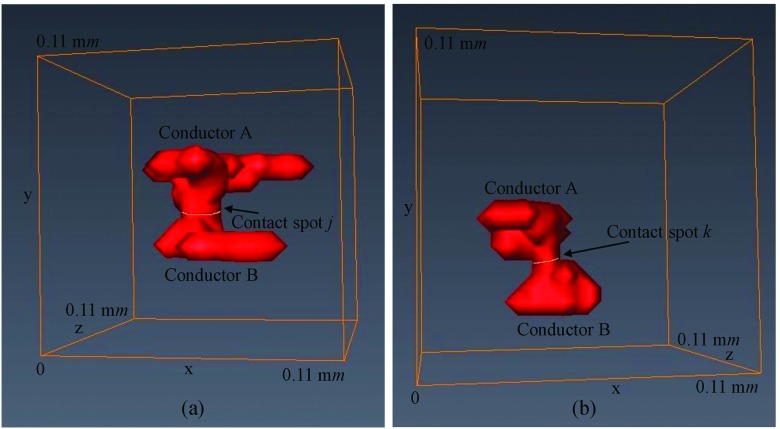
(a) Contact asperity *j* and (b) Contact asperity *k*.

Fig. 11a, Fig. 12a and Fig. 12b, illustrate the structures of contact spots *i, j* and *k* to the two conductors of the contact system of Fig. 7 respectively. Each of these contact spots is also illustrated in the 2D contact map of Fig. 9a. In addition, the 3D contact asperities of contact spots *i, j* and *k* are illustrated in Fig. 11c, Fig. 13a and Fig. 13b respectively. The 3D contact asperities of Fig. 11c, Fig. 13a and Fig. 13b are the closed-up view of the corresponding 3D contact asperities of Fig. 10.

**FIG. 14.**
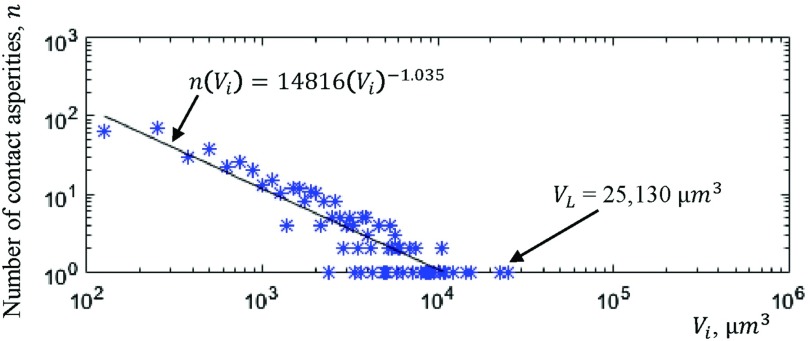
Contact asperity volume distribution.

Fig. 14 illustrates the graph of 3D contact asperity volume distribution. The data are taken from the 3D contact asperities of Fig. 10. The volume of each 3D contact asperity is defined as the sum of voxels within the 3D contact asperity and multiplied by 125 μ*m*^3^ (volume of voxel). The smallest volume of 3D contact asperity indicated on the graph of Fig. 14 is 125 μ*m*^3^ which is the resolution of the technique (1 voxel = 125 μ*m*^3^). The largest 3D contact asperity indicated are several thousand μ*m*^3^ in the volume (*V*_*L*_ = 25,130 μ*m*^3^). The number of 3D contact asperities, *n* is counted to be 466.

**FIG. 15.**
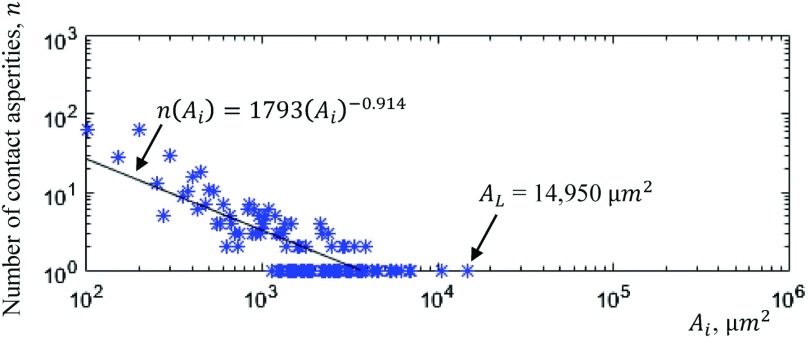
Contact asperity surface area exposed to air distribution.

Fig. 15 illustrates the 3D contact asperity surface area distribution. The data are taken from the 3D contact asperities of Fig. 10. The surface area of each 3D contact asperity is defined as the sum of pixels of the 3D contact asperity which are exposed to air and multiplied by 25 μ*m*^2^ (pixel area). The smallest surface area of 3D contact asperity indicated on the graph of Fig. 15 is 100 μ*m*^2^ which is four times the resolution of the technique (1 pixel = 25 μ*m*^2^). The largest 3D contact asperity indicated are several thousand μ*m*^2^ in the volume (*A*_*L*_ = 14,950 μ*m*^2^).

## V. DISCUSSION

### A. Visualization method

The results show that the X-ray CT is a powerful visualization method for viewing the contact interface of a contact system without needing to dismantle it. The data acquired using this method with pixel resolution of 5 μ*m* × 5 μ*m* and voxel resolution of 5 μ*m* × 5 μ*m* × 5 μ*m* give the ability to examine and process in order to investigate different characteristics which occur in the contact interface and its extension.

The resolution is a very important factor for the calculation and visualization methods. For example, for a coarse measurement (e.g. 100 μ*m*) of resolution, only a few asperities of large curvature are visualized while for smaller measurement (e.g. 0.1 μ*m*) of resolution, more asperities of smaller curvature are visualized.^1,2^ The smallest resolution which can be obtained by the current facility is 3 μ*m*. This depends on the sample dimensions and X-ray admittance of the sample materials. The resolution of 5 μ*m* obtained in this work is the optimum that could be achieved with the sample configuration used. However, it should be noted that the CAT* developed and implemented within a suite of tools in this work can be used with data of finer resolution and for different visualization methods which are producing 2D cross-section slice images. The different visualization methods, for example the MRI^8,9,43,44^ and Magnetic Resonance Force Microscopy (MRFM)^45–48^ can be used with the suite of tools developed in this paper.

### B. Contact analysis approach

A contact analysis approach is developed and introduced in this paper for 3D visualization of the contact asperities for contact systems. This approach uses the 2D cross-section slice images of the 3D source model which have been processed as described in previous work^14^ in order to build the 3D contact source model. From this 3D contact source model, the 3D contact asperities have been visualized. The contact analysis approach consists of contact analysis techniques (CATA, CATV and CATAC) which are developed in MATLAB using the Image Processing Toolbox.

The selection of the limits of the 3D contact source model in the y-direction (the distance between the limits of the 3D contact source model in the y-direction equals the number of x-z cross-section slices) depends on the number of slice asperities in each x-z cross-section slice above and below the 3D constriction asperities map. The first x-z cross-section slice of the 3D contact source model is the first slice which has only one slice asperity below the 3D constriction asperities map while the last x-z cross-section slice of the 3D contact source model is the first slice which has only one slice asperity above the 3D constriction asperities map. The first and last x-z cross-section slices of the 3D contact source mode are the connections of all contact asperities. This can be clearly seen in the schematic 3D contact source model of Fig. 4a.

In previous work^11^ the 3D contact map of an electrical contact interface is developed demonstrating the 3D nature of the contact. In this work, the 3D contact map is used in order to demonstrate the structures of 3D contact asperities. Fig. 8 illustrates the 3D contact map of the 250 *V*, 16 *A* rated AC single pole rocker switch which is converted into the 2D contact map as presented in Fig. 9a. The 2D contact map is used as the reference x-z contact slice to make a comparison of each contact spot of the x-z contact slice with the spots of each x-z cross-section slice of the 3D contact source model of the 250 *V*, 16 *A* rated AC single pole switch. The reason for this comparison is to visualize the full structures above and below of each contact spot to the two conductors of the contact system. This result is described by the visualization of three different contact spots, *i*, *j* and *k* which are selected randomly from the 2D contact map of Fig. 9a. The full structures above and below of these *i*, *j* and *k* contact spots to the two conductors are illustrated in Fig. 11a, Fig. 12a and Fig. 12b respectively. A closed-up view of the full structures above and below of contact spot *i* to the two conductors which are illustrated in Fig. 11a is presented in Fig. 11b as volume of interest around the contact spot *i*. The volume of interest around the contact spot *i* consists of the stack of x-z cross-section slice images which are cropped from the coordinates of the yellow dash-box of Fig. 9b. The volume of interest around the contact spot *i* of Fig. 11b is selected to typing an asperity for visualisation in order to show that the actual 3D contact asperity is a part of this volume of interest (see Fig. 11c). For the actual 3D contact asperities visualization which are part of the full structures above and below of each contact spot Eqs. (6), (7) and (8) are used as described in Section III.B.

### C. 3D contact asperities findings

Fig. 10 shows the 3D contact asperities which are visualized using the contact analysis approach. These 3D contact asperities present the asperity structures above and below the contact spots of Fig. 9a to the two conductors of the contact system. In addition, the 3D contact asperities are shown to have different sizes, shapes and their contact spots vary. It is important to mention that the total number of 3D contact asperities is equal to the total number of contact spots (467). Fig. 10 illustrates only the 466 3D contact asperities of the contact system. This is because the largest 3D contact asperity of the contact system (the largest contact spot in the x-z contact slice of Fig. 9a) is not presented as it hides the other 3D contact asperities.

Fig. 11c, Fig. 13a and Fig. 13b illustrate a closed-up view of the actual 3D contact asperities *i*, *j* and *k* of Fig. 10 with their contact spots *i*, *j* and *k* respectively. The volume of each of these *i*, *j* and *k* 3D contact asperities is calculated to be 10,500 μ*m*^3^, 17,875 μ*m*^3^ and 8,000 μ*m*^3^ respectively. This volume, is defined as the sum of voxels within the 3D contact asperity and multiplied by 125 μ*m*^3^ (the volume of voxel). The contact spot area of each of these *i*, *j* and *k* 3D contact asperities is calculated to be 25 μ*m*^2^, 75 μ*m*^2^ and 25 μ*m*^2^ respectively. This area, is defined as the sum of pixels within the 3D contact spot of 3D contact asperity and multiplied by 25 μ*m*^2^ (the area of pixel). In addition, the surface area of each of these *i*, *j* and *k* 3D contact asperities is calculated to be 4,950 μ*m*^2^, 7,825 μ*m*^2^ and 3,975 μ*m*^2^ respectively. The surface area of each 3D contact asperity is defined as the sum of pixels of the 3D contact asperity which are exposed to air and multiplied by 25 μ*m*^2^ (the area of pixel). The surface area of the 3D contact asperityτ with τ∈[1,n] (*n*, is the total number of 3D contact asperities which is also equal with the total number of contact spots) that is exposed to air is described from Eq. (9). Where *A*_*sur*_, is the total surface area of 3D contact asperityτ and *A*_*A*_ and *A*_*B*_ are the surface areas of the top and bottom of the of 3D contact asperityτ respectively.

$$A_{\tau }=A_{sur}-A_{A}-A_{B}.$$ (9)

The graph of Fig. 14 illustrates the 3D contact asperity volume distribution for the 3D contact asperities of Fig. 10. The volume of the largest 3D contact asperity $\left(V_{L}\right)$ of Fig. 10 is 25,130 μ*m*^3^ while the majority of 3D contact asperities have a volume equal with 150 *μm*^3^ (59 3D contact asperities in number). Moreover, it can be seen that the graph consists of two regimes. The first where the 3D contact asperity volume distribution follows a power law relationship with a slope of 1.035 and the second is for a small number of particular large 3D contact asperities in volume with a slope approximately equal to zero. In addition, the sum of the volume of 3D contact asperities gives the total volume of 3D contact asperities which is found to be *V* = 833,375 *μm*^3^ (the total volume of 3D contact asperities of Fig. 10). The total volume of 3D contact asperities is given from Eq. (10). Where *V*τ** is the total volume of contact asperity*τ* with τ∈[1,n] (*n*, is the total number of 3D contact asperities).

$$V=\sum_{\tau =1}^{n}V_{\tau }.$$ (10)

The graph of Fig. 15 illustrates the 3D contact asperity surface area exposed to air distribution for the 3D contact asperities of Fig. 10. The surface area exposed to air of the largest 3D contact asperity (*A*_*L*_) of Fig. 10 is 14,950 μ*m*^2^ while the majority of 3D contact asperities have a surface area exposed to air equal with 200 μ*m*^2^ (61 3D contact asperities in number). Moreover, it can be seen that the graph consists of two regimes. The first where the 3D contact asperity surface area exposed to air distribution follows a power law relationship with a slope of 0.914 and the second is for a small number of particular large 3D contact asperities in volume with a slope approximately equal to zero. In addition, the sum of the surface area exposed to air of the 3D contact asperities gives the total surface area of the 3D contact asperities exposed to air which is found to be *A* = 407,925 μ*m*^2^ (total surface area exposed to air of the 3D contact asperities of Fig. 10). The total surface area exposed to air of 3D contact asperities is given from Eq. (11).

$$A=\sum_{\tau =1}^{n}A_{\tau }.$$ (11)

The calculations of volume and surface area which is exposed to air of the 3D contact asperities may be useful by researchers which are interested in the characterisation of contact systems under different factors which play important role on their reliability. These factors are the normal-force (force perpendicular to the surface) and the general contact design, the wear and the environmental and electrical parameters.^49^

## VI. CONCLUSION

The X-ray CT method is used to visualize the contact system of a 250 *V*, 16 *A* rated AC single pole rocker switch without the need of dismantling the sample. A contact analysis approach is developed and introduced in this paper to show in a 3D visualization the contact asperities of a given contact system. This approach is based on the cross-section slices of the contact system which are acquired from the X-ray CT and are processed using Contact Analysis Techniques, CAT* which are developed and implemented with a suite of tools developed in MATLAB and Image Processing Toolbox. These techniques can be used for any contact system and for any value of resolution.

The contact asperities consist of voxel or voxels which represent the 3D microstructures of the contact system and it is found that they have different sizes, shapes and their contact spots are vary. Moreover, the volume and surface area exposed to air of each of the 3D contact asperities of the 250 *V*, 16 *A* rated AC single pole rocker switch are calculated and presented with their distributions. In the analysis of both distributions (3D contact asperities volume and surface area exposed to air) it was observed that the 3D contact asperities followed a power law relationship.
